# Shoes with active insoles mitigate declines in balance after fatigue

**DOI:** 10.1038/s41598-020-58815-9

**Published:** 2020-02-06

**Authors:** Jeongin Moon, Prabhat Pathak, Sudeok Kim, Se-gon Roh, Changhyun Roh, Youngbo Shim, Jooeun Ahn

**Affiliations:** 1 Department of Physical Education, Seoul National University, Seoul, Republic of Korea; 2 Samsung Advanced Institute of Technology, Samsung Electronics Co., Ltd., Suwon, Republic of Korea; 3 Institute of Sport Science, Seoul National University, Seoul, Republic of Korea

**Keywords:** Neuroscience, Physiology, Engineering

## Abstract

Fatigue can induce postural instability and even lead to falls. However, most current methods to delay or reduce fatigue require long preparatory time, or large and expensive equipment. We propose a convenient method to alleviate postural instability due to fatigue. We paid attention to that fatigue and aging share similar neurophysiological deterioration of sensory-motor function. Considering that stochastic resonance via sub-sensory mechanical vibration increases postural stability in the elderly, we propose that sub-sensory insole vibration reduces the negative effect of fatigue on postural control. We performed experiments with 21 young and healthy adult participants, and demonstrated that insole vibration compensates for the loss of balance ability due to fatigue. The sub-sensory insole vibration restored both the area of center of pressure and the complexity of the time series of the motor output after fatigue to the pre-fatigue levels. The insole units generating the vibration were completely concealed in shoes and controlled by a smart phone. This compact implementation contrasts with the cumbersome procedure of current solutions to fatigue-induced postural instability.

## Introduction

Various motor tasks including daily activities and sports competitions induce fatigue. Repetitive contractions of specific muscle decrease maximal voluntary contraction strength, and general muscular exercises such as hiking, running, and cycling increase energy expenditure levels beyond the lactic acid accumulation threshold^1^. In both cases, fatigue impairs somatosensory receptor and muscle function^2–6^. Multiple studies have quantified the effect of fatigue on postural stability, and found that fatigue diminishes postural control ability and causes higher variability of joint movement and unintentional body tremor^7–12^. These negative effects of fatigue elevate the risk of fall regardless of age and lower the quality of life^13–15^. Thus, effective methods to counteract the negative effects of fatigue on postural stability need to be explored.

To date, delay or reduction of the negative effects of fatigue on motor functions has mostly depended on use of preventive measures. Consumption of dietary supplements such as caffeine, beta-alanine, and arginine prior to exercise delays the onset of fatigue in both young and elderly adults^16–18^. Application of light-emitting diode and therapeutic laser also delays the onset of fatigue and enhances recovery from muscular damage^19,20^. However, application of these procedures is not simple; it requires months of preparation, vexatious treatment procedures, and/or expensive equipment.

We considered stochastic resonance (SR) as a way to reduce the negative effects of fatigue on postural stability. SR refers to a phenomenon in which a particular frequency of the original signal resonates with a part of noise^21^. Collins *et al*. have shown that noise can increase somatosensory perception when SR amplifies the necessary signal to exceed the detection threshold^22^. More specifically, application of sub-threshold mechanical vibration enhances the sensitivity of degraded somatosensory systems in the elderly and patients with peripheral neuropathy, resulting in an increase in postural stability^23–26^. In the case of young healthy adults, application of SR through electrical stimulation on vestibular system improves postural control^27^.

We hypothesize that sub-threshold mechanical vibration generated by an active insole can counteract the degradation of postural stability after fatigue. From a neurophysiological perspective, similarities exist between the effects of fatigue and those of aging on postural stability. Fatigue reduces muscular force generation by disturbing ionic exchange in the muscle due to the production of metabolites^4^. In addition, repetitive muscle contraction induces the depletion of neurotransmitters and retards sensory reflexes^28^. Similarly, aging leads to a decrease in the number of alpha motor neurons, and deterioration in the sensitivity of sensory reflexes^29–31^. Consequently, both fatigue and aging impede sensory reflexes and motor control^32–34^. Therefore, considering both the benefit of sub-threshold mechanical vibration to the elderly and the similarity of the neuromuscular symptoms due to fatigue and aging, the sub-threshold mechanical vibration might be a viable way to compensate for negative functional changes due to fatigue.

Our hypothesis is plausible but not obvious. Although sub-threshold mechanical vibration has been observed to benefit the elderly and patients with peripheral neuropathy, the efficacy of sub-threshold mechanical vibration in enhancing postural stability in young healthy adults is controversial. Priplata *et al*. claimed that sub-threshold mechanical vibration shows significant utility for young adults^24^, but others suggested the contrary^35–37^.

**Figure 1:**
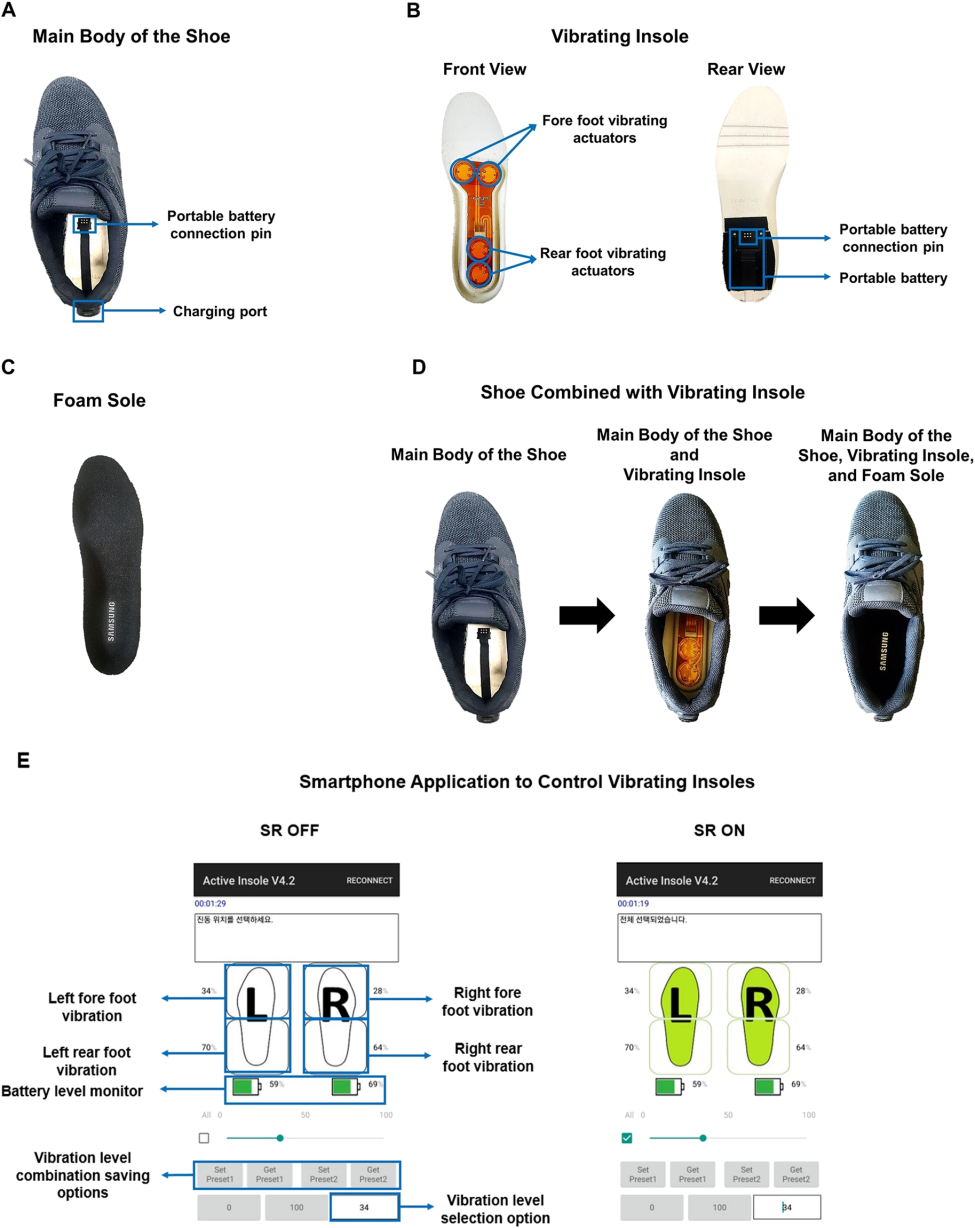
Overview of the active insoles, shoes and the smartphone application used to control the vibration. (**A**) The main body of the shoe. The shoe contains a charging port linked to a portable battery connection pin. (**B**) The front and the rear view of the vibrating insoles. The front view shows the position of the pairs of actuators. The actuators at the top and the bottom apply mechanical vibration to the metatarsal head (fore foot), and to the heel (rear foot), respectively. The rear view shows the position of the portable battery and the connection pin. (**C**) The foam which is placed on top of the vibrating insole as a shock absorber. (**D**) The sequence of attachment of components. (**E**) The overview of the smartphone application which controls the vibrating insoles. The application allows separate control of the amplitude of the vibration for the fore and rear parts of left and right foot. Users can save and load combinations of the vibration amplitude levels, and monitor the battery level. The area for each part of the feet turns green on the application screen when the vibration is turned on.

In this study, we introduce shoes with active insoles that generate vibration whose amplitude is wirelessly controlled by a smart phone (Fig. 1). To test the feasibility of the proposed compact technology, we quantify balancing ability of young healthy adults before and after fatigue with and without sub-sensory vibration applied to the sole by the active insole. We additionally investigate the change in the level of complexity of the time series of the motor output due to fatigue and vibration.

## Results

**Figure 2:**
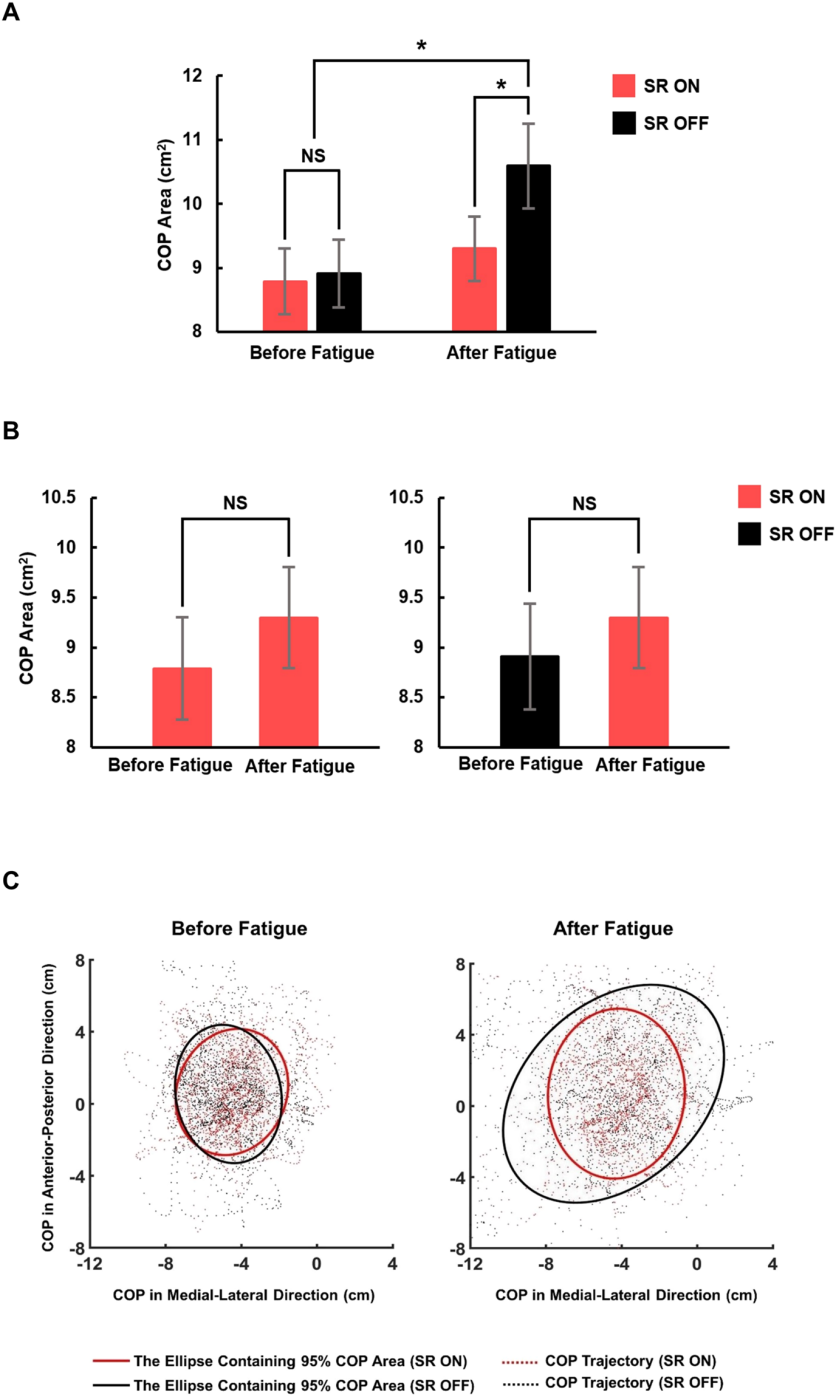
The center of pressure (COP) ellipse areas in the four experimental conditions. (**A**) The comparison of the mean COP ellipse areas of 21 participants under all four experimental conditions (SR ON and SR OFF conditions before and after fatigue). (**B**) The comparison of the mean COP ellipse areas of 21 participants under SR ON and OFF conditions before fatigue with SR ON condition after fatigue. The error bars denote standard errors. *Statistically significant difference (p < 0.05); NS: no statistically significant difference (p > 0.05). Pair-wise comparison during one-way repeated measures analysis of variance revealed that the COP ellipse areas in the SR OFF after fatigue condition was significantly larger than the areas in the other 3 experimental conditions. No significant difference was observed among COP ellipse areas in the SR ON and SR OFF conditions before fatigue, and that in the SR ON condition after fatigue. **(C)** The representative ellipses of containing 95% COP areas in the SR ON and SR OFF conditions before and after fatigue.

Each participant performed a balancing task under the following four conditions: SR ON (using sub-threshold insole vibration) and SR OFF (without vibration) conditions before and after inclined loaded walking that induces general fatigue. Consulting the quantification method devised by Oliveira *et al*.^38^, we quantified balancing ability by measuring the 95% confidence interval ellipse area of the center of pressure (COP). The mean and standard error (SE) of the ellipse areas of 21 participants under the four experimental conditions are shown in Fig. 2A. Two-way repeated measures analysis of variance (ANOVA) showed main effects of vibration and fatigue on the COP ellipse area (vibration: F_[1,20]_ = 7.838, p = 0.011, and fatigue: F_[1,20]_ = 8.093, p = 0.010), and a significant interaction between the vibration and fatigue (F_[1,20]_ = 5.419, p = 0.031). Thus, we additionally performed one-way repeated measures ANOVA, and the result showed a main effect of the experimental conditions on the COP ellipse area (F_[3,60]_ = 7.345, p < 0.001). Pair-wise comparisons revealed that the COP ellipse areas in the SR OFF condition after fatigue were significantly larger than COP ellipse areas in the other three experimental conditions, whereas no significant differences were observed among COP ellipse areas in the SR ON condition after fatigue, SR ON condition before fatigue, and SR OFF condition before fatigue. In particular, ANOVA results showed that the insole vibration did not affect COP ellipse area before fatigue, whereas, after fatigue, the insole vibration significantly reduced the COP ellipse areas (p = 0.022) to the level similar to that before fatigue (Fig. 2B). The data set of the COP trajectories and 95% confidence interval ellipse of a single representative participant in the four different conditions are shown in Fig. 2C.

**Figure 3:**
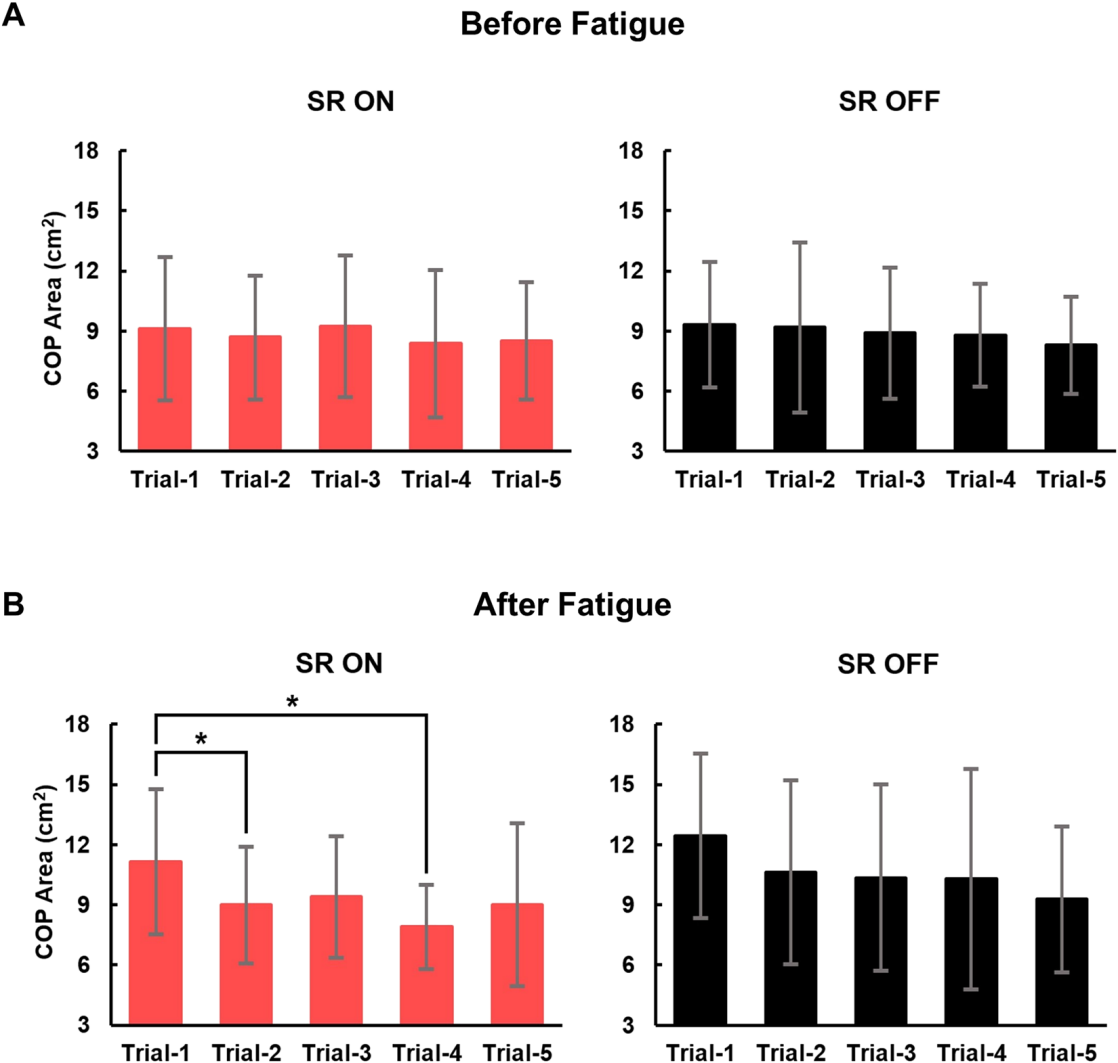
The center of pressure (COP) ellipse areas versus the sequential order of trials in the four experimental conditions. (**A)** The comparison of the mean COP ellipse areas for 21 participants under the SR ON and SR OFF conditions before fatigue. **(B)** The comparison of the mean COP ellipse areas for 21 participants in the SR ON and SR OFF conditions after fatigue. The error bars denote standard deviations. *Statistically significant difference (p < 0.05). Pairwise comparisons during one-way repeated measures analysis of variance revealed that there were no significant differences in COP areas between trials in the SR ON and SR OFF conditions before fatigue and SR OFF condition after fatigue. In the SR ON condition after fatigue, the mean COP ellipse area of trial-1 was significantly larger than those of trial-2 and trial-4.

The effect of the execution order on the balancing performance was also analyzed. The sequences of SR ON and SR OFF were randomized, but the number of total trials of each condition was matched. The mean and standard deviation (SD) of the ellipse areas of 21 participants versus the sequential order of trials in each experimental condition are shown in Fig. 3. We performed one-way repeated measures ANOVA to investigate whether the COP ellipse areas changed due to the sequential order of trials. A significant main effect of trial order on COP ellipse area was observed in only the SR ON after fatigue condition (F_[4,80]_ = 0.399, p = 0.809 during SR ON before fatigue; F_[4,80]_ = 0.625, p = 0.646 during SR OFF before fatigue; F_[4,80]_ = 4.762, p = 0.002 during SR ON after fatigue; and F_[4,80]_ = 1.944, p = 0.111 during SR OFF after fatigue). Only during SR ON after fatigue, the COP ellipse areas in trial-1 were significantly larger than the areas in trial-2 (p = 0.045) and trial-4 (p = 0.005), and no statistically significant difference was observed between any other pair of trials under any experimental condition.

**Figure 4:**
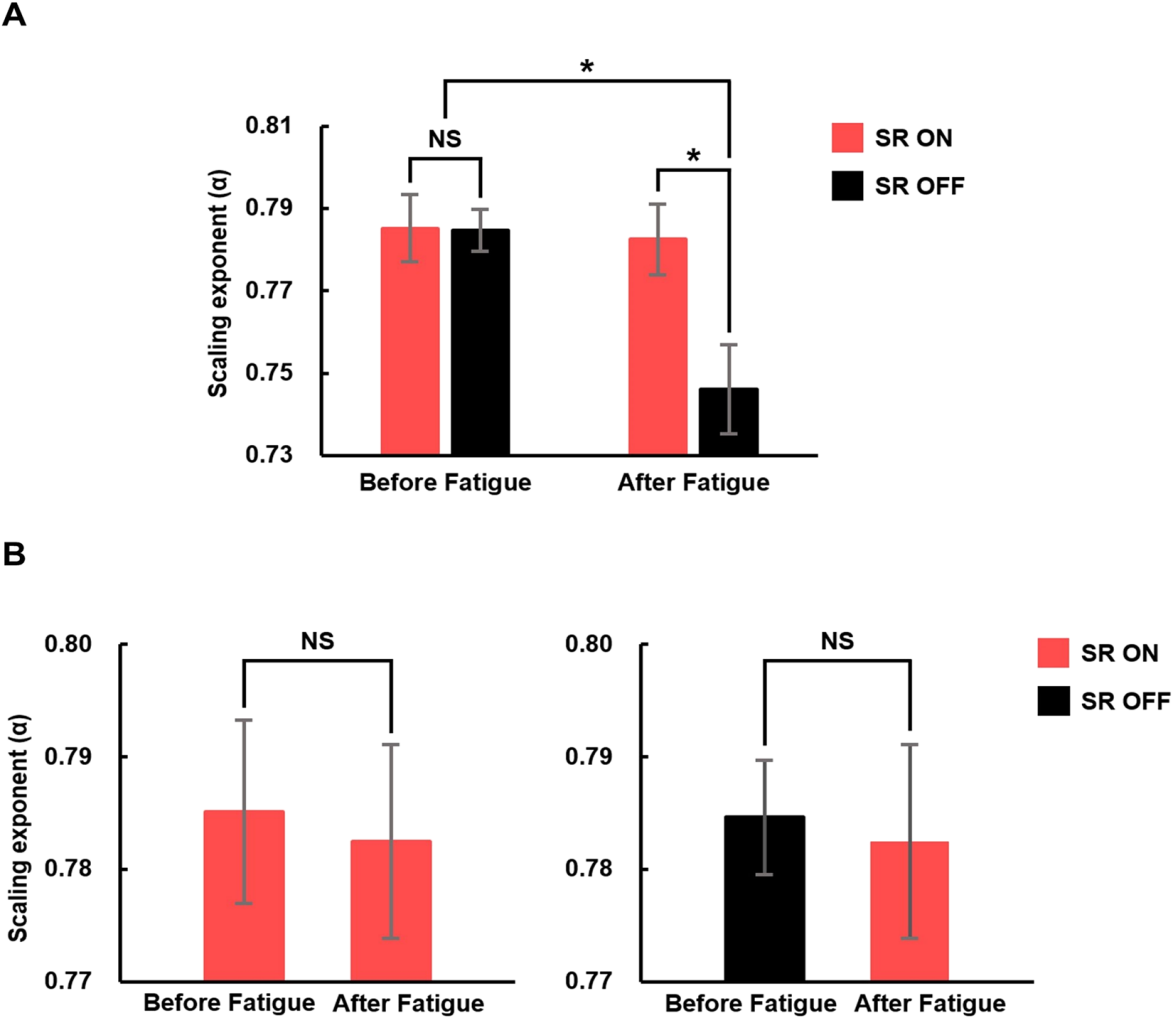
The scaling exponents (α) obtained from the time series of the center of pressure (COP) displacement. (**A**) The comparison of the mean α of 21 participants under all four experimental conditions (SR ON and SR OFF conditions before and after fatigue). (**B**) The comparison of the mean α of 21 participants under SR ON and OFF conditions before fatigue with SR ON condition after fatigue. The error bars denote standard errors. *Statistically significant difference (p < 0.05); NS: no statistically significant difference (p > 0.05). Pair-wise comparison during one-way repeated measures analysis of variance revealed that the values of α in the SR OFF after fatigue condition was significantly smaller (and farther from unity) than the values of α in the other 3 experimental conditions; without the sub-sensory vibration, fatigue significantly decreased the complexity of the time series of COP. No significant difference was observed among the values of α in the SR ON and SR OFF conditions before fatigue, and that in the SR ON condition after fatigue.

The structural complexity of the time series of COP was quantified using scaling exponent α obtained from detrended fluctuation analysis (DFA)^39^. A time series is considered similar to white, pink or Brownian noise when α is close to 0.5, 1, or 1.5, respectively, and the structure of the time series is considered maximally complex when α is equal to unity^40^. The mean and standard error (SE) of the values of α of 21 participants under the four experimental conditions are shown in Fig. 4. Two-way repeated measures ANOVA showed main effects of vibration and fatigue on α (vibration: F_[1,20]_ = 7.907, p = 0.011, and Fatigue: F_[1,20]_ = 6.422, p = 0.020), and a significant interaction between the vibration and fatigue (F_[1,20]_ = 11.529, p = 0.003). Thus, we additionally performed one-way repeated measures ANOVA, and the result showed a main effect of the experimental conditions on the values of α (F_[3,60]_ = 8.074, p < 0.001). Pair-wise comparisons revealed that the values of α in the SR OFF condition after fatigue were significantly smaller than the values of α in the other three experimental conditions, whereas no significant differences were observed among the values of α in the SR ON after fatigue, SR ON before fatigue, and SR OFF before fatigue. In particular, ANOVA concluded that the insole vibration did not affect the complexity of the time series before fatigue, whereas, after fatigue, the vibration restored the complexity to the level similar to that before fatigue.

**Figure 5:**
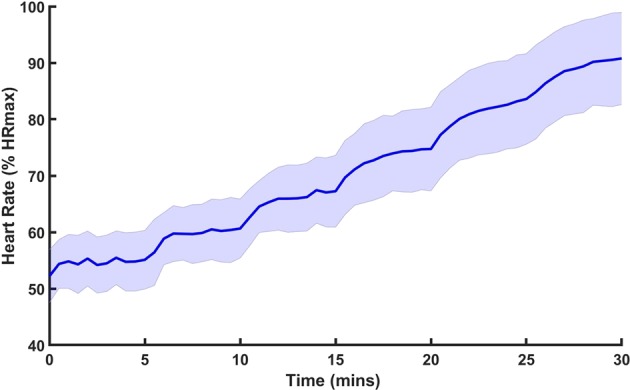
The heart rate (HR) during the inclined loaded walking. The solid blue line represents the mean HR of 21 participants and semi-transparent interval represents the SD interval during the inclined loaded walking. HR continued increasing during the fatigue-inducing task. For each participant, HR_MAX_ was calculated using the following formula: 208–0.7 × age. The final HRs of all participants exceeded 70% HR_MAX_.

For each participant, the maximum heart rate (HR_MAX_) was calculated as 208–0.7 × age^41^. The time course of the mean and standard deviation (SD) of heart rate (HR) relative to HR_MAX_ during the inclined loaded walking task is shown in Fig. 5. For all 21 participants, HR continued to increase throughout the walking task. The final HR of all participants exceeded 70% of HR_MAX_, beyond which lactate typically begins to accumulate^42^.

## Discussion

The current study explores the use of sub-sensory mechanical vibration on the sole to counteract declined balancing ability following a fatigue-inducing task. A large COP area indicates degraded postural stability, and our results showed that fatigue significantly increased the COP area during one-legged balancing, but the insole vibration reduced the COP area almost to the pre-fatigue level (Fig. 2). This suggests that sub-sensory insole vibration is a viable intervention to improve balancing ability after fatigue even for young healthy adults.

The change in the structure of the time series of COP additionally supports the positive effect of the sub-sensory vibration. The small variability observed in many stereotyped human behaviors cannot be modeled as uncorrelated noise. Long-range correlations or fractal-like features are frequently observed in various “healthy” human behaviors including walking, heart beating and brain activity, whereas such rich complexity vanishes due to aging or neurological disorders^43,44^. These observations have led to the notion that structural complexity of the time series of the motor output is a sign of healthy flexibility^45^; a flexible, or healthy sensory-motor system is expected to show rich complexity or a long range correlation, whereas a declined system is expected to lose the complexity. A scaling index can be used to assess the structural complexity of resultant motor output; a scaling exponent α far from unity indicates decreased complexity or flexibility^40,46^. Our results clearly showed that fatigue pushed the values of α of the COP time series away from unity, whereas the sub-sensory mechanical vibration restored the degraded complexity to the pre-fatigue level (Fig. 4).

The improvement of postural stability in fatigued young healthy adults by applying sub-sensory vibration can be attributed to a temporary increase in the sensory threshold induced by fatigue and subsequent compensation by SR. The perceptive threshold of sensory organs increases from the normal level immediately after heavy exhaustive exercise^47^. Sub-sensory vibration can engender SR and amplify the input signals that the sensory system should detect beyond the increased threshold. The amplification of input signals also results in the restoration of the complexity and increase in functional capability of the postural control system^40^. Consequently, noisy sub-sensory mechanical vibration can compensate for the loss of sensitivity induced by fatigue.

Conversely, before fatigue, no significant difference between COP ellipse area in the SR ON and that in the SR OFF conditions was shown. Without fatigue, the scaling exponent did not change due to the sub-sensory vibration either. The observation that the sub-sensory vibration did not enhance balancing before fatigue is consistent with the results of multiple previous studies. It has been reported that SR enhances postural stability in the elderly, whereas no significant effect is observed when the same noise is applied to young and healthy control group^35,36^. Zarkou *et al*. also demonstrated that SR improves balancing of children with cerebral palsy but did not affect the balancing of their typically developing peers^37^. These results indicate that SR shows efficacy only for individuals whose sensory function is not intact. Dependence of the effect of SR on the threshold can plausibly explain these observations. Although some studies regard SR as a distinct phenomenon from previously reported negative masking^48^, it is also frequently regarded as a phenomenon in which noise is used as a suitable masker or pedestal^22^. The effect of negative masking depends on the threshold; the masker does not increase sensitivity when the threshold is too low relative to the applied noise^49^. Similarly, Greenwood *et al*. reported that the effectiveness of SR depends on the noise intensity and the difference between the threshold and the sensory signal^50^. If an individual’s sensory system already has a low threshold or high sensitivity as that seen in unfatigued young and healthy individuals, SR may not enhance balancing ability. On the other hand, the fatigued group, the elderly and patients with peripheral neuropathy have high sensory thresholds and are much more likely to benefit from SR^48,51^.

However, it is plausible that the increase in sensory threshold is neither the entire underlying mechanism of the postural instability after fatigue, nor the only reason for the efficacy of the sub-sensory vibration. Fatigue affects not only the somatosensory system but also the neurotransmitter pathways, central nervous system (CNS), and motor function^52^. Considering the type and the intensity of the fatigue-inducing task adopted in this study and the resultant high HR observed during the experiment (Fig. 5), the participants are expected to undergo fatigue effects on various systems besides the sensory organs. Nevertheless, the current study showed no significant difference between balancing ability after fatigue in the SR ON condition and that before fatigue; the sub-sensory mechanical vibration applied to the sole of the participant restored sensory-motor performance after fatigue to a similar performance level observed before fatigue (Figs. 2B and 4B). This suggests that the sub-sensory vibration might restore function of the various components in the sensory-motor system rather than only inducing SR and amplifying the input signals that the sensory system should detect.

Multiple studies actually support the notion that sub-sensory mechanical vibration not only induces SR but also affects other parts of the sensory-motor system. Manjarrez *et al*. reported that noise applied to sensors increases the sensitivity of the CNS as well as the sensitivity of the sensors of adult cats^53^; they demonstrated that sensory stimuli increase the sensitivity of CNS to specific sensory signals by increasing the field potential of the CNS area processing that particular sensory information. Mendez-Balbuena *et al*. reported that sub-sensory vibration increases accuracy during a finger force control task, and suggested that the sub-sensory vibration enhances neuronal synchronization at the spinal, cortical, and corticospinal levels^54^. The contribution of sub-sensory stimuli to various parts of the sensory-motor system provides plausible explanation of how the sub-sensory vibration can almost restore the balancing ability after fatigue to that before fatigue.

To increase the reliability of the measurement, we evaluated balance ability in five trials in each of the four experimental conditions. However, the effect of fatigue may be attenuated by recovery as the trial number increases. To minimize any artifact due to the recovery after fatigue, the balancing task was performed immediately after the fatigue-inducing task, and the performance order of SR ON and SR OFF conditions was randomized. Figure 3 shows that the deterioration in balance ability was not immediately restored after fatigue. In general, the performance order of trial conditions did not significantly affect the COP ellipse area except in two trials in the SR ON after fatigue condition.

We determined the walking speed and the load weight of the fatigue-inducing task, based on the standard hiking speed and the average weight of young and healthy men and women. The exercise protocol was standardized based on the participant’s gender, and the participants, depending on their gender, were asked to walk on the same treadmill with the same belt speed following the same profile of the slope with the same load. However, the amount of physiological fatigue might vary across participants depending on their physical strength and endurance. In this initial study, we aimed only to investigate the effects of sub-sensory vibration before and after fatigue, and the HR of all participants exceeded 70% HR_MAX_, beyond which lactate accumulation typically begins^42^. Further systematic research on the relationship between the amount of physiological fatigue and efficacy of SR might need to be considered in the future.

We did not record electromyography and did not monitor the amount of fatigue induced by inclined loaded walking in specific muscles that need to be activated during the balancing task. Accordingly, we cannot conclude the extent that peripheral muscle fatigue is responsible for the deterioration of the balancing performance. We confined the scope of this study to investigating the efficacy of sub-sensory vibration under general fatigue from usual real life activities. The inclined loaded walking adopted in this study is not a local muscular exercise but a whole body muscular exercise, which imitates intense hiking. This type of general muscular exercise typically deteriorates the sensory proprioceptive, exteroceptive information and/or their integration, and/or decreases the muscular system efficiency^8,9^.

In our study, the sample sizes of male and female were 17 and 4, respectively. Due to this difference in the sample size, the results from both genders could not be compared statistically in this initial study. To investigate any possible difference between men and women in their response to the sub-sensory vibration to the soles, further experiments with more systematic recruitment of participants are required.

The results of this study have important practical implications. Fatigue is highly correlated with postural instability^1,14^, and postural instability induced by fatigue increases the risk of fall even in young and healthy adults^13^. Our study demonstrates the efficacy of sub-sensory vibration in enhancing postural stability after fatigue, which can lower the risk of fall. We also illustrate that this effective sub-sensory vibration can be implemented compactly. In this study, the vibration was applied by embedding the active insole units in shoes. The rechargeable battery and the actuator were all inside the shoes, and the vibration was wirelessly controlled by a smart phone; the implementation required only a smart phone and shoes without any wire outside the active insole. In contrast, most current methods to reduce or delay the negative effects of fatigue require either a long preparation period or unportable equipment. The efficacy in improving balance and the simple implementation of the active insole vibration unit suggest the use of the devised system as a convenient intervention to compensate for deterioration in balance due to fatigue.

## Methods

### Participants

Twenty-one healthy young adults (17 men and 4 women; age: 27 ± 4.48 years; height: 172.81 ± 7.56 cm; weight: 69.90 ± 11.01 kg) participated in the study. This sample size is larger than the necessary size, which was calculated as 11 in Supplementary Methods. The participants had no known neuromuscular and cardiovascular disorders. More information about the participants is provided in Supplementary Table S1. All aspects of this study conformed to the principles and guidelines described in the Declaration of Helsinki, and the Institutional Review Board of Seoul National University approved this study. Participants provided informed, written consent prior to participation.

### Equipment

Shoes embedded with active insole units were used to provide mechanical vibration to the soles of the feet (Fig. 1). The active insole units consisted of four piezoelectric actuators (Disc Benders-Bimorphs, model no. 20–2225, American Piezo Ceramics; diameter: 21 ± 0.30 mm; thickness: 0.36 ± 0.10 mm; maximum frequency: 1–350 Hz; drive voltage: 15 V), two each at the front and rear sections of the insole. The distance between the two actuators at each section was 1 cm and the minimum distance between actuators at the front and rear sections of the insoles was 8 cm. This allowed us to deliver mechanical vibration to the metatarsal head (fore foot) and heel (rear foot) of the left foot and right foot separately. The components of the electrical circuit were placed at the center of the rubber shell of the insole and were insulated with a coating material to avoid electrical contact with the participant’s foot. Custom-built smartphone software allowed us to control vibration amplitude wirelessly via Bluetooth technology. Vibration amplitude for each foot section (fore and rear foot) and limb (left and right foot) could be controlled independently.

A HR monitor (OH1, Polar, USA) was used to measure heart rate during the inclined loaded walking task. A military backpack was used as the load bearing apparatus. A treadmill (Gait analysis FDM-TDSL-3i, Zebris, Germany; belt length: 150 cm; belt width: 50 cm; maximum incline: 15%; maximum speed: 24 km/hr) was used for the walking task. A single force platform (FP6090-15-2000, Bertec, Columbus, OH, USA) was used to collect COP data in anterior-posterior (COP_AP_) and medial-lateral (COP_ML_) directions at a sampling frequency of 100 Hz.

### Experimental procedure

#### Determination of sensory threshold

During the sensory threshold determination procedure, the participants were instructed to sit in an upright position and maintain equal weight on each foot. Although the sensory threshold is higher in a standing position than a seated position^55^, maintaining one’s balance while standing requires incessant shifting of weight and unintentional joint movement that might result in unreliable measurement of the sensory threshold^56^. Further, the sensory thresholds for the fore foot and rear foot and for the left and right feet were determined separately as the sensory thresholds differ depending on the part of the sole^55^. Vibration amplitude was applied in an incremental order to detect individual sensory thresholds; participants were instructed to report once they sensed the vibration. For each of the four foot areas, the estimation procedure was repeated 3 times, and the mean of the three estimations was chosen as the sensory threshold for each specific part of the sole, respectively. The order of sensory threshold measurement for each foot area was randomly determined per each participant.

#### Static balancing task

**Figure 6:**
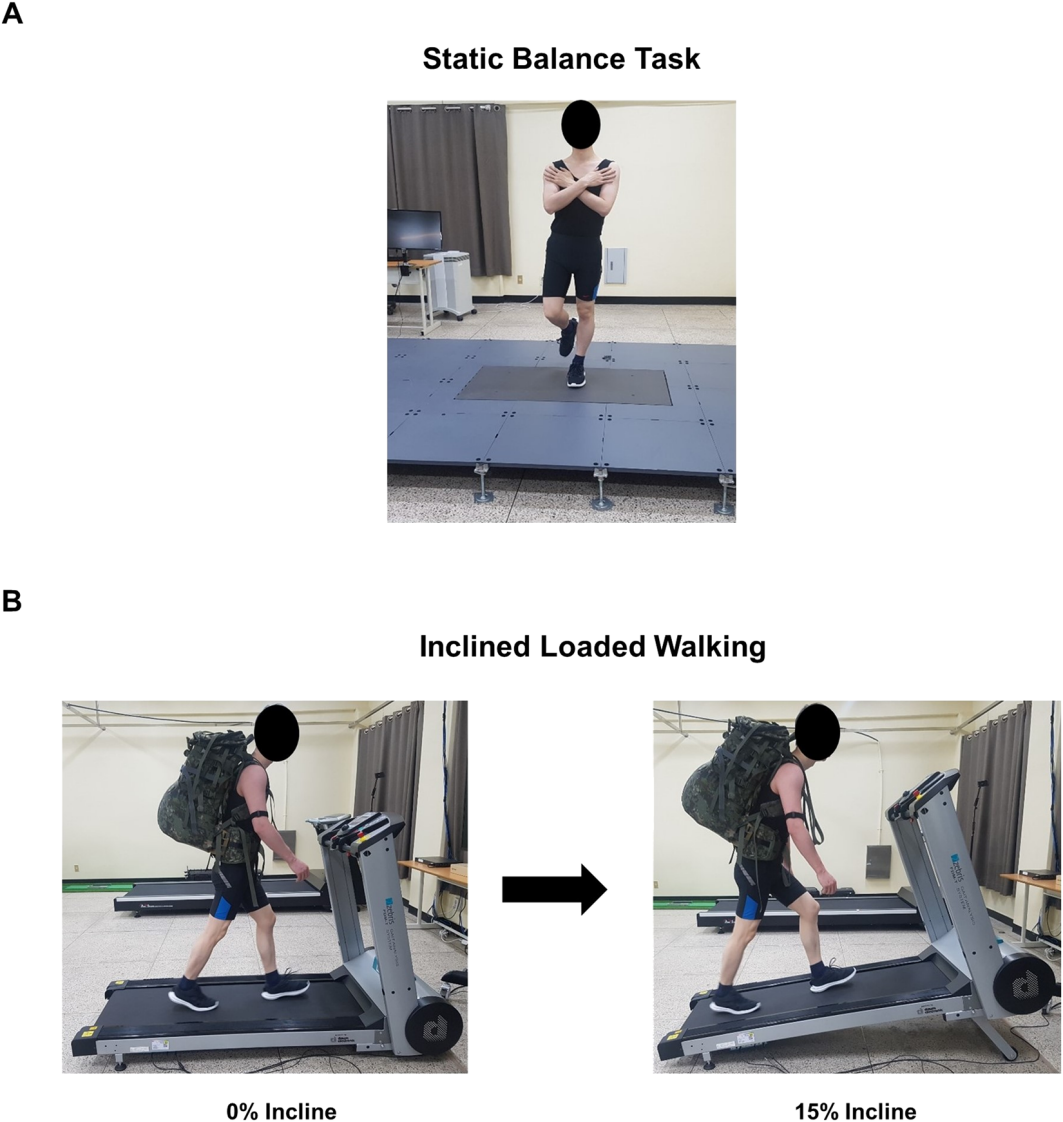
The static balancing task and the inclined loaded walking task. (**A)** During the single-legged static balancing task, the participants stood on a force platform. They were requested to cross their arms and place their hands on the shoulders. **(B)** During the inclined loaded walking task, the participant walked on the treadmill at a constant speed (men: 3.8 km/hr; women: 3.5 km/hr) carrying a loaded backpack (men: 20 kg; women: 15 kg). The participant walked for a total of 30 minutes and the incline was increased by 3% every 5 minutes (beginning at 0% and reaching 15% incline by the end).

The single-legged static balancing task was performed before and after performing the fatigue-inducing task. The amplitude of sub-sensory vibration was set at 90% of the sensory threshold. The participants were instructed to stand on the force platform with their non-dominant leg for 30 seconds. They were requested to cross their arms and place their hands on their shoulders (Fig. 6A). Each participant performed 10 trials each before and after fatigue; of each set of 10 trials, 5 were performed in the SR ON condition and 5 were performed in the SR OFF condition. The order of trials with the SR ON and SR OFF conditions was randomized. A rest of 40 seconds was given between trials. Participants were instructed to report if they could sense vibration during the experiment, but no participant reported sensing vibration during the experiment.

#### Inclined loaded walking task

The participants were provided with comfortable attire to wear during the inclined loaded walking. The HR monitor was strapped on the upper arm. The weight of the backpack was 20 kg and 15 kg for male and female participants, respectively; these weights correspond to 30% of the standard body weight of the national population of Republic of Korea^57^. The treadmill belt speeds for male and female participants were 3.8 km/hr and 3.5 km/hr, respectively, throughout the inclined loaded walking. Participants walked for 30 minutes after a 2-minute warm-up and the incline was increased by 3% every 5 minutes (beginning at 0% and reaching 15% at the end of the protocol) (Fig. 6B). It was followed by a cool-down period of 1 minute during which the participants walked at 0% incline.

### Data processing

COP data during the initial five seconds of the 30-second balancing task were removed to allow time for the participants to acquire a stable posture. The data during the remaining 25 seconds were then filtered using zero-lag, 4^th^ order low-pass filter with a cut-off frequency of 10 Hz. The COP area was calculated using the 95% confidence interval ellipse area obtained from COP_AP_ and COP_ML_^38^.

Then, the time series of magnitude of COP displacement was obtained as

1 $$D(j)=\sqrt{{(CO{P}_{ML}(i)-CO{P}_{ML}(i-1))}^{2}+{(CO{P}_{AP}(i)-CO{P}_{AP}(i-1))}^{2}},$$

where, *i* = 2, …, *L*, and *L* is the total length of the time series of COP_AP_ and COP_ML_. Detrended fluctuation analysis (DFA) was used to extract the scaling exponent α^39^. First, cumulatively sum *D* to obtain an integrated time series, *D*_*int*_(*k*), as

2 $${D}_{int}(k)=\mathop{\sum }\limits_{i=1}^{k}\,(D(j)-{D}_{avg}),$$

where *D*(*j*) and *D*_*avg*_ are the *j*^th^ coordinate and the average of the magnitude of displacement, and *k* = 1, 2, …, *N*, where *N* is the total length of *D*_*int*_(*k*). The time series *D*_*int*_(*k*) is then divided into non-overlapping windows of length *n* samples. For each window size *n*, a local least squares line fit is calculated in every window, and define the fitted value as *D*_*det*_(*k*). The average fluctuation of *D*_*int*_(*k*) with respect to the locally best fit line is calculated for each window size *n* as

3 $$F(n)=\sqrt{\frac{1}{N}\mathop{\sum }\limits_{k=1}^{N}{[{D}_{int}(k)-{D}_{det}(k)]}^{2}}.$$

The slope of the least square fit line relating log *F*(*n*) and log *n* is the scaling exponent α, which becomes 0.5 for white noise, 1.5 for Brownian noise, and a value between 0.5 and 1 for time series with long range correlations. Following the suggestion in previous studies^39,46^, the window size increased from the initial value of 4 to the final value of *N*/4 with the increment of the number of samples occupying 50 ms, which was 5 in this study.

### Statistical analysis

In the first set of statistical analyses, two-way repeated measures ANOVA was used to evaluate (1) significant differences in mean COP ellipse areas and the scaling exponents for 21 participants depending on the experimental condition performed (SR ON and SR OFF conditions before and after fatigue), and (2) the interaction between vibration and fatigue. Once a significant interaction between the two factors was found, one-way repeated measures ANOVA was additionally performed. In the second set of statistical analyses, one-way repeated measures ANOVA was used to assess effects of the trial order on the mean COP ellipse areas of 21 participants under each experimental condition. For both sets of statistical analyses, Bonferroni correction was selected as a post-hoc test for multiple pairwise comparisons. Mauchly’s sphericity test was performed when the test was applicable, and the results confirmed that no ANOVA violated the assumption of sphericity in this study. The results of Mauchly’s sphericity test are summarized in Supplementary Table S2. The level of statistical significance was set at p < 0.05.

## Supplementary information

Supplementary information.

## Data Availability

All data sets generated and/or analyzed during the current study are available from the corresponding author on reasonable request.
