# Supplementary material for: Shoes with active insoles mitigate declines in balance after fatigue
Source: Sci Rep. 2020 Feb 6;10:1951. doi: 10.1038/s41598-020-58815-9 (PMC7004992; doi:10.1038/s41598-020-58815-9)
Supplement: Supplementary file 1 — Supplementary information. [file 41598_2020_58815_MOESM1_ESM.pdf]

SUPPLEMENTARY INFORMATION FOR:
Shoes with active insoles mitigate declines in balance after fatigue

Jeongin Moon[1], Prabhat Pathak[1], Sudeok Kim[1], Se-gon Roh[2], Changhyun Roh[2], Youngbo Shim[2] and Jooeun Ahn[1, 3*]

[1]Department of Physical Education, Seoul National University, Republic of Korea

[2]Samsung Advanced Institute of Technology, Samsung Electronics Co., Ltd.

[3]Institute of Sport Science, Seoul National University, Republic of Korea

*Correspondence should be addressed to Prof. Jooeun Ahn

ahnjooeun@snu.ac.kr

Tel: +82-2-880-7785

SUPPLEMENTARY METHODS

Sample size calculation

Consulting a previous meta-analysis study that addressed the effect of stochastic noise on a single-leg standing balance[1] we set the effect size as 0.49. Further, we set the correlation among the repeated measures and p-value for statistical significance as 0.5, and 0.05, respectively[2]. The G*Power software[3] calculated the required sample size as 11.

References

1. Woo, M. T. *et al.* Effects of different lower-limb sensory stimulation strategies on postural regulation—A systematic review and meta-analysis. *PLOS ONE* **12**, e0174522 (2017)
2. Hur, P., Pan, Y.-T. & DeBuys, C. free energy principle in Human postural control System: Skin Stretch feedback Reduces the entropy. *Sci. Rep.* **9**, 1-11 (2019)
3. Faul, F., Erdfelder, E., Buchner, A. & Lang, A.-G. Statistical power analyses using G* Power 3.1: Tests for correlation and regression analyses. *Behav. Res. Methods* **41**, 1149-1160 (2009).

Supplementary Table S1. Additional information of the participants

	Sex	Age	Height	Weight	Race	% HR (0min)	% HR (30min)
SUB-1	M	27	187	80	Asian	53.41	88.84
SUB-2	M	37	177	86	Asian	55.46	103.24
SUB-3	M	25	176	74	Asian	52.49	85.04
SUB-4	M	31	178	73	Asian	55.82	94.47
SUB-5	M	29	170	60	Asian	53.28	98.56
SUB-6	M	27	177	75	Asian	55.00	97.30
SUB-7	M	32	171	82	Asian	54.96	95.37
SUB-8	M	23	180	55	Asian	56.28	98.49
SUB-9	M	24	179	68	Asian	40.79	89.44
SUB-10	M	22	173	77	Asian	57.63	95.53
SUB-11	M	34	168	67	Asian	47.23	90.66
SUB-12	M	34	170	79	Asian	53.75	106.95
SUB-13	M	24	177	81	Asian	59.10	92.05
SUB-14	M	30	182	77	Asian	46.52	73.26
SUB-15	M	24	175	80	Asian	50.73	79.50
SUB-16	M	25	170	65	Asian	52.06	87.63
SUB-17	M	26	174	72	Asian	50.05	91.15
SUB-18	W	25	168	63	Asian	47.24	82.41
SUB-19	W	25	163	54	Asian	47.77	79.79
SUB-20	W	23	156	50	Asian	47.94	90.67
SUB-21	W	20	158	50	Asian	58.79	86.09

Heights are in cm and weights are in kg. %HR (0min) and %HR (30min) are the % ratio of the heart rate to the maximum heart rate at the beginning and the end of the loaded walking task, respectively. For each participant, the maximum heart rate is calculated as 208 – 0.7 × age.

Supplementary Table S2. Results of Mauchly's test of sphericity

Variables	Results
Effect of experimental conditions on mean COP ellipse areas of 21 participants	χ^2 (5) = 8.331, p = 0.139
Change in COP ellipse areas of 21 participants due to sequential order of trials for each of the experimental conditions.	SR ON Before Fatigue: χ^2 (9) = 12.155, p = 0.206<br>SR OFF Before fatigue: χ^2 (9) = 12.873, p = 0.170<br>SR ON After Fatigue: χ^2 (9) = 9.913, p = 0.359<br>SR OFF After Fatigue: χ^2 (9) = 6.589, p = 0.681
Effect of experimental conditions on mean alpha exponents of 21 participants	χ^2 (5) = 6.347, p = 0.275
